# Supplementary material for: High Uric Acid Levels in Acute Myocardial Infarction Provide Better Long-Term Prognosis Predictive Power When Combined with Traditional Risk Factors
Source: J Clin Med. 2022 Sep 21;11(19):5531. doi: 10.3390/jcm11195531 (PMC9573253; doi:10.3390/jcm11195531)
Supplement: Supplementary file 1 [file jcm-11-05531-s001.zip › jcm-1871091-supplementary.pdf]

Supplemental materials

Table S1. Baseline Clinical and Medication Characteristics (After IPW Adjustment).

	Original Cohort					After IPW-Adjusted			
	Total (*n* = 5888)	Normal uric acid (*n* = 4326)	High uric acid (*n* = 1562)	*p*-value	absolute SMD	Normal uric acid	High uric acid	*p*-value	absolute SMD
Clinical characteristics									
Age, year	64.0 ± 12.7	63.6 ± 12.4	65.4 ± 13.3	<0.001	0.141	64.0 ± 12.4	63.8 ± 13.5	0.589	0.017
Age ≥65 year	3010 (51.1)	2119 (49.0)	891 (57.0)	<0.001	0.162	3010.7 (50.8)	3045.2 (52.1)	0.419	0.025
Male	4141 (70.3)	3085 (71.3)	1056 (67.6)	0.007	0.081	4183.7 (70.6)	4159.4 (71.2)	0.703	0.012
BMI, kg/m2	24.1 ± 3.2	24.0 ± 3.2	24.3 ± 3.4	0.022	0.069	24.1 ± 3.2	24.1 ± 3.3	0.921	0.003
BMI >25 kg/m2	2069 (35.1)	1485 (34.3)	584 (37.4)	0.032	0.064	2060.1 (34.8)	2064.6 (35.3)	0.713	0.011
DM	2232 (37.9)	1584 (36.6)	648 (41.5)	0.001	0.1	2265.3 (38.2)	2215.9 (37.9)	0.827	0.007
Hypertension	4182 (71.0)	3008 (69.5)	1174 (75.2)	<0.001	0.126	4212.8 (71.1)	4146.9 (70.9)	0.906	0.004
History of dyslipidemia	1371 (23.3)	1013 (23.4)	358 (22.9)	0.716	0.012	1365.3 (23.0)	1348.7 (23.1)	0.985	0.001
History of Stroke	472 (8.0)	326 (7.5)	146 (9.3)	0.027	0.065	480.2 (8.1)	485.5 (8.3)	0.811	0.007
Current smoker	2280 (38.7)	1724 (39.9)	556 (35.6)	0.003	0.088	2284.4 (38.6)	2276.9 (39.0)	0.8	0.008
Previous MI	259 (4.4)	187 (4.3)	72 (4.6)	0.688	0.014	278.8 (4.7)	241.7 (4.1)	0.401	0.028
Previous PCI	456 (7.7)	327 (7.6)	129 (8.3)	0.406	0.026	479.4 (8.1)	464.0 (7.9)	0.862	0.006
Previous CABG	32 (0.5)	22 (0.5)	10 (0.6)	0.685	0.017	34.0 (0.6)	29.1 (0.5)	0.723	0.01
Atrial fibrillation on baseline ECG	224 (3.8)	144 (3.3)	80 (5.1)	0.002	0.089	223.7 (3.8)	218.5 (3.7)	0.942	0.002
eGFR <30 mL/min/1.73 m2	412 (7.0)	183 (4.2)	229 (14.7)	<0.001	0.362	461.4 (7.8)	433.2 (7.4)	0.631	0.014
Chronic liver disease	59 (1.0)	43 (1.0)	16 (1.0)	1	0.003	58.2 (1.0)	62.9 (1.1)	0.765	0.009
Chronic lung disease	153 (2.6)	101 (2.3)	52 (3.3)	0.043	0.06	152.7 (2.6)	162.1 (2.8)	0.692	0.012
Cancer	198 (3.4)	142 (3.3)	56 (3.6)	0.626	0.017	197.7 (3.3)	212.8 (3.6)	0.595	0.016
LVEF	52.7 ± 11.6	53.4 ± 11.1	50.5 ± 12.6	<0.001	0.248	52.5 ± 11.7	52.7 ± 11.8	0.63	0.015
LVEF ≤35%	505 (8.6)	288 (6.7)	217 (13.9)	<0.001	0.24	533.5 (9.0)	511.3 (8.7)	0.757	0.009
ST-segment elevation MI	3095 (52.6)	2313 (53.5)	782 (50.1)	0.023	0.068	3098.9 (52.3)	3090.8 (52.9)	0.72	0.011
ACEF score	1.4 ± 0.6	1.3 ± 0.6	1.5 ± 0.7	<0.001	0.371	1.4 ± 0.6	1.4 ± 0.6	0.529	0.02
Medication at discharge									
Aspirin	5793 (98.4)	4258 (98.4)	1535 (98.3)	0.761	0.012	5825.1 (98.3)	5758.2 (98.5)	0.638	0.014
Clopidogrel	5151 (87.5)	3761 (86.9)	1390 (89.0)	0.04	0.063	5189.8 (87.6)	5126.2 (87.7)	0.935	0.003
Ticagrelor	331 (5.6)	240 (5.5)	91 (5.8)	0.73	0.012	327.6 (5.5)	321.7 (5.5)	0.97	0.001
Prasugrel	371 (6.3)	301 (7.0)	70 (4.5)	0.001	0.107	370.4 (6.3)	361.8 (6.2)	0.94	0.003
Potent P2Y12 inhibitor	702 (11.9)	541 (12.5)	161 (10.3)	0.024	0.069	698.0 (11.8)	683.6 (11.7)	0.932	0.003
Beta-blocker	5131 (87.1)	3824 (88.4)	1307 (83.7)	<0.001	0.137	5160.3 (87.1)	5086.4 (87.0)	0.922	0.003
ACEi or ARB	4443 (75.5)	3333 (77.0)	1110 (71.1)	<0.001	0.137	4473.1 (75.5)	4462.9 (76.3)	0.514	0.02
Oral anticoagulant	154 (2.6)	97 (2.2)	57 (3.6)	0.004	0.083	152.0 (2.6)	155.0 (2.7)	0.85	0.005
Statin at discharge	5638 (95.8)	4154 (96.0)	1484 (95.0)	0.102	0.049	5672.0 (95.7)	5594.9 (95.7)	0.955	0.002

Data are presented as the n (%) for categorical variables and as the mean ± standard deviation for continuous variables. PS indicates propensity-score; IPW, inverse probability weighted; SMD, standardized mean differences; BMI, body mass index; DM, diabetes mellitus; MI, myocardial infarction; CABG, coronary artery bypass graft; ECG, electrocardiography; eGFR, estimated

glomerular filtration rate; LVEF, left ventricle ejection fraction; ACEi, angiotensin converting enzyme inhibitors; ARB, angiotensin II receptor blockers.

Table S2. Baseline Laboratory and Angiographic Characteristics (After IPW Adjustment).

	Total (*n* = 5888)	Original Cohort				After IPW-Adjusted			
		Normal uric acid (*n* = 4326)	High uric acid (*n* = 1562)	*p*-value	absolute SMD	Normal uric acid	High uric acid	*p*-value	absolute SMD
Laboratory findings									
Uric acid, mg/dL	5.6 ± 4.0	4.3 ± 1.4	9.1 ± 6.1	<0.001	1.071	4.3 ± 1.4	9.2 ± 6.9	<0.001	0.991
Uric acid, μmol/L	(333.1 ± 237.9)	(255.8 ± 83.3)	(541.3 ± 362.8)			(255.8 ± 83.3)	(547.2 ± 410.4)		
CK-MB, peak, ng/mL	130.0 ± 753.8	124.4 ± 267.3	145.2 ± 1394.5	0.558	0.021	124.3 ± 276.3	140.0 ± 1175.9	0.539	0.018
Hemoglobin, mg/dL	13.4 ± 2.2	13.5 ± 2.1	13.1 ± 2.5	<0.001	0.154	13.4 ± 2.1	13.4 ± 2.3	0.562	0.018
Creatinine, mg/dL	1.2 ± 1.2	1.1 ± 1.0	1.5 ± 1.5	<0.001	0.344	1.3 ± 1.7	1.3 ± 1.0	0.862	0.007
high-sensitivity CRP, mg/dL	5.3 ± 18.5	5.1 ± 18.1	5.7 ± 19.6	0.286	0.032	5.4 ± 19.1	4.8 ± 17.3	0.205	0.038
Total cholesterol, mg/dL	176.8 ± 43.2	176.7 ± 42.6	177.1 ± 45.0	0.772	0.009	175.7 ± 43.0	178.4 ± 43.7	0.043	0.063
Triglyceride, mg/dL	122.2 ± 89.4	119.2 ± 84.4	130.3 ± 101.7	<0.001	0.119	122.7 ± 90.7	122.5 ± 87.5	0.923	0.003
High-density lipoprotein, mg/dL	40.6 ± 10.8	41.0 ± 10.9	39.4 ± 10.7	<0.001	0.149	40.5 ± 10.8	40.5 ± 11.3	0.894	0.005
Low-density lipoprotein, mg/dL	112.3 ± 37.1	112.5 ± 37.0	111.8 ± 37.6	0.52	0.019	111.7 ± 37.1	113.0 ± 37.5	0.247	0.036
Angiographic characteristics									
Multivessel disease	3198 (54.3)	2259 (52.2)	939 (60.1)	<0.001	0.16	3218.0 (54.3)	3139.7 (53.7)	0.698	0.012
Left main PCI	230 (3.9)	156 (3.6)	74 (4.7)	0.057	0.057	228.1 (3.9)	220.0 (3.8)	0.876	0.005
Left anterior descending PCI	3515 (59.7)	2595 (60.0)	920 (58.9)	0.471	0.022	3580.0 (60.4)	3399.9 (58.2)	0.14	0.046
Left circumflex PCI	1589 (27.0)	1188 (27.5)	401 (25.7)	0.183	0.041	1634.6 (27.6)	1482.3 (25.4)	0.106	0.051
Right coronary artery PCI	2364 (40.1)	1718 (39.7)	646 (41.4)	0.269	0.033	2368.0 (40.0)	2392.3 (40.9)	0.535	0.019
Total stent number	1.6 ± 0.9	1.6 ± 0.9	1.6 ± 0.9	0.103	0.048	1.6 ± 0.9	1.6 ± 0.9	0.968	0.001
Total stent length	37.6 ± 25.0	37.2 ± 24.8	38.6 ± 25.5	0.05	0.058	37.5 ± 25.2	37.7 ± 24.8	0.796	0.008
Bifurcation PCI with two stents	91 (1.5)	57 (1.3)	34 (2.2)	0.025	0.066	90.3 (1.5)	86.2 (1.5)	0.884	0.004
Long stenting >60mm	283 (4.8)	209 (4.8)	74 (4.7)	0.937	0.004	298.0 (5.0)	254.3 (4.3)	0.295	0.032
Restenosis lesion	91 (1.5)	67 (1.5)	24 (1.5)	1	0.001	90.8 (1.5)	76.7 (1.3)	0.542	0.019
Ostial lesion	232 (3.9)	158 (3.7)	74 (4.7)	0.07	0.054	227.6 (3.8)	265.9 (4.5)	0.25	0.035
Second generation DES	3499 (59.4)	2616 (60.5)	883 (56.5)	0.007	0.08	3511.4 (59.3)	3477.0 (59.5)	0.893	0.004

Data are presented as the n (%) for categorical variables and as the mean ± standard deviation for continuous variables. PS indicates propensity-score; IPW, inverse probability weighted; SMD, standardized mean differences; CK-MB, creatine kinase muscle brain; CRP, C-reactive protein; PCI, percutaneous coronary intervention; DES, drug-eluting stent.

Table S3. Predictive Factors for Mortality According to multivariable Cox Regression Analysis.

	Unadjusted		Multivariable-adjusted	
	HR (95% CI)	*p*-value†	HR (95% CI)	*p*-value†
High uric acid	1.69 (1.52,1.88)	< 0.001	1.18 (1.05,1.32)	0.005
Age ≥65 years	5.01 (4.4,5.7)	< 0.001	3.47 (3.01,4)	< 0.001
LVEF ≤35%	3.19 (2.79,3.65)	< 0.001	2 (1.74,2.29)	< 0.001
Atrial fibrillation	1.88 (1.53,2.32)	< 0.001	1.6 (1.28,1.99)	< 0.001
eGFR <30 mL/min/1.73 m^2	5.25 (4.6,5.99)	< 0.001	1.52 (1.25,1.86)	< 0.001

History of stroke	2.17 (1.87,2.51)	< 0.001	1.43 (1.23,1.67)	< 0.001
Diabetes mellitus	1.77 (1.6,1.97)	< 0.001	1.23 (1.1,1.37)	< 0.001
Creatinine	1.22 (1.19,1.24)	< 0.001	1.08 (1.04,1.12)	< 0.001
ACEi or ARB	0.48 (0.43,0.53)	< 0.001	0.55 (0.49,0.62)	< 0.001
Hemoglobin	0.75 (0.73,0.76)	< 0.001	0.88 (0.86,0.91)	< 0.001
Body mass index	0.54 (0.48,0.61)	< 0.001	0.75 (0.67,0.85)	< 0.001
Clopidogrel	1.63 (1.31,2.02)	< 0.001	0.6 (0.39,0.93)	0.021
Triglyceride	1 (1,1)	< 0.001	1 (1,1)	0.003
HDL cholesterol	0.99 (0.98,0.99)	< 0.001	0.99 (0.99,1)	0.003
Potent P2Y12 inhibitor	0.49 (0.38,0.63)	< 0.001	0.5 (0.3,0.85)	0.01
LDL cholesterol	0.99 (0.99,0.99)	< 0.001	1 (1,1)	0.05
ST-segment elevation MI	0.68 (0.61,0.76)	< 0.001	0.92 (0.82,1.02)	0.11
Male	0.57 (0.51,0.63)	< 0.001	1.1 (0.98,1.25)	0.113
Prasugrel	0.31 (0.2,0.46)	< 0.001	0.68 (0.41,1.13)	0.135
Chronic lung disease	1.92 (1.49,2.46)	< 0.001	1.2 (0.93,1.55)	0.155
History of dyslipidemia	0.74 (0.65,0.85)	< 0.001	1.09 (0.94,1.26)	0.27
hypertension	1.43 (1.27,1.62)	< 0.001	1.04 (0.92,1.18)	0.542
Current smoker	0.48 (0.43,0.54)	< 0.001	1.03 (0.9,1.18)	0.657
Multivessel disease	1.44 (1.3,1.61)	< 0.001	1.02 (0.91,1.14)	0.712
Oral anticoagulant	1.41 (1.06,1.88)	0.019	0.95 (0.71,1.28)	0.75

†p value from Cox regression. High uric acid is defined as >6.5mg/dL for male and >5.8mg/dL for female. HR indicates hazard ratio; CI, confidence interval; LVEF, left ventricular ejection fraction; ACEi, angiotensin converting enzyme inhibitors; ARB, angiotensin II receptor blockers; eGFR, estimated glomerular filtration rate; HDL, high density lipoprotein; LDL, low density lipoprotein; MI, myocardial infarction.

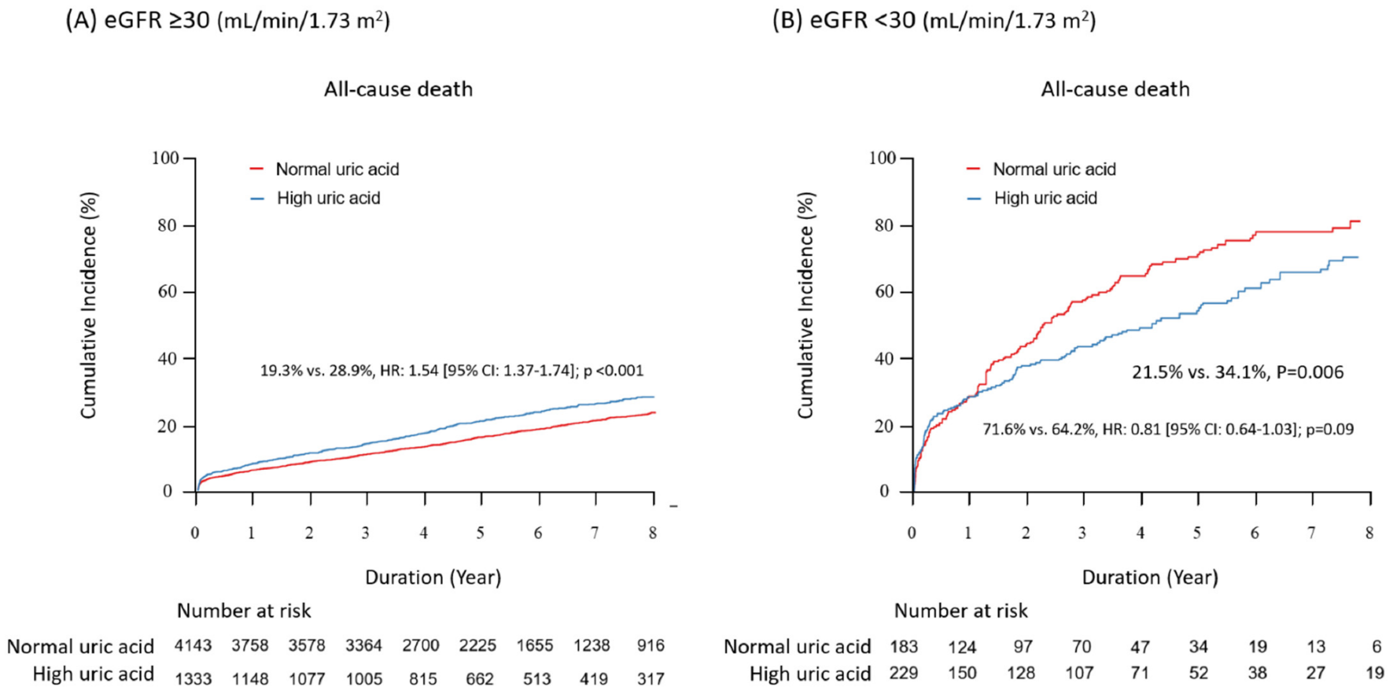

Figure S1. Kaplan-Meier Curve With Cumulative Hazards of All-cause Death Compared According to the Uric Acid Level in the Subgroups of (A) eGFR ≥30 mL/min/1.73 m^2 and (B) eGFR <30 mL/min/1.73 m^2.
